# Proteins Secreted via the Type II Secretion System: Smart Strategies of *Vibrio cholerae* to Maintain Fitness in Different Ecological Niches

**DOI:** 10.1371/journal.ppat.1003126

**Published:** 2013-02-21

**Authors:** Aleksandra E. Sikora

**Affiliations:** Department of Pharmaceutical Sciences, College of Pharmacy, Oregon State University, Corvallis, Oregon, United States of America; Duke University Medical Center, United States of America

## Introduction

Many Gram-negative bacteria use the type II secretion (T2S) pathway to deliver proteins that contribute to disease in humans, animals, and plants [1]. *Vibrio cholerae*, the causative agent of the life-threatening diarrheal disease cholera, utilizes the T2S system for translocation of at least 19 proteins, including the large hexameric protein cholera toxin (Table S1) [1], [2]. The release of cholera toxin is predominantly responsible for the voluminous diarrhea in afflicted patients. The T2S machinery consists of 12 Eps (**e**xtracellular **p**rotein **s**ecretion) proteins and prepilin peptidase PilD. The secretion of the T2S substrates (exoproteins, cargo proteins) is a two-step process including their translocation via the inner membrane by the Sec or Tat pathway and subsequent transport of folded and/or oligomeric cargo proteins by the T2S into the extracellular milieu. The structure and function of the individual constituents of the T2S machinery in *V. cholerae* have been addressed in many elegant studies and recently reviewed [1]. This review focuses rather on the T2S substrates, highlighting their importance in *V. cholerae* pathophysiology, functional interactions, and mechanisms regulating their expression.

## Cholera Toxin: The Key Virulence Factor Secreted by the T2S

**Figure 1:**
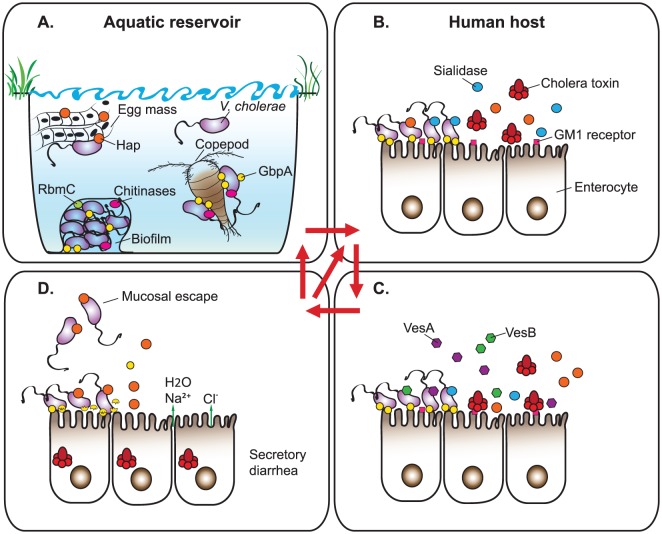
The T2S substrates facilitate survival and fitness of *V. cholerae* in its dual life cycle. (A) The aquatic stage of the *V. cholerae* life cycle. GbpA facilitates attachment of the bacteria to various biotic (e.g., copepods) and abiotic chitinous surfaces. Chitinases and HapA enable the utilization of chitin and insect egg masses as nutrient sources, respectively, while RbmC aids in maintaining the structural integrity of biofilms. (B, C, D) The human host phase of the *V. cholerae* life cycle. (B) In the initial stages of infection, *V. cholerae* utilizes GbpA along with other factors to colonize the epithelial lining of the small intestine. The mucinase complex (including HapA and sialidase) hydrolyzes the mucus in the gut lumen and removes sialic acid from higher order gangliosides to reveal the receptor for cholera toxin (GM1). (C) Cholera toxin binds to the GM1 receptors on the surface of enterocytes. Proteases HapA, VesA, and VesB cleave the protease-sensitive loop connecting the A_1_ and A_2_ peptides within the A subunit of cholera toxin. (D) Cholera toxin enters the enterocytes and, after a cascade of reactions, induces a secretory diarrhea. During the late stage of infection, HapA degrades GbpA. Subsequently, *V. cholerae* detaches from the intestinal epithelium, penetrates through the fluid-filled lumen, and prepares to enter the aquatic environment or another human host. Please refer to the text for complementary information.

Pathogenic *V. cholerae* persist in natural aquatic reservoirs and infect the human host via consumption of contaminated water or food (Figure 1). The bacteria passage through the gastrointestinal tract and, after colonization of the small intestine, produce and subsequently secrete cholera toxin via the T2S system [3], [4]. Cholera toxin is the major virulence factor of *V. cholerae*, as the symptoms of the disease can be recapitulated upon administration of purified toxin to human volunteers. The 84-kDa cholera toxin is a prototypical AB_5_ enterotoxin composed of an A subunit noncovalently bound to a pentamer of B subunits. The A subunit is comprised of two polypeptide chains, A_1_ and A_2_, which are responsible for the toxin's ADP-ribosyltransferase activity and bridging the A and B subunits, respectively. In order to exert its enzymatic activity, the A subunit requires proteolytic processing at Arg192 prior to entering intestinal epithelial cells. The proteolytic cleavage of cholera toxin can be accomplished by proteases endogenous to the gut lumen and secreted by *V. cholerae* (Figure 1) [2], [5], [6]. The B subunits recognize GM1 ganglioside receptors on the surface of enterocytes and facilitate the binding of cholera toxin to the cell membrane. Subsequent retrograde transport on lipid rafts delivers toxin as a fully folded protein complex from the plasma membrane into the endoplasmic reticulum, where protein disulfide isomerase binds, unfolds, and disassembles the A_1_ domain from the rest of the toxin complex. After these events, the A_1_ subunit exits the endoplasmic reticulum via retrotranslocation and ADP-ribosylates the heterotrimeric GTPase G_sα_. The modified G_sα_ activates adenylate cyclase, which raises the levels of cAMP and induces the Cl^−^ secretory response, followed by flow of Cl^−^ and water into the intestinal milieu causing profuse watery diarrhea, the hallmark of cholera [3], [4]. The “rice-water” stools released by cholera patients contain a large number of vibrios in a transient hyperinfectious state, which, if ingested by subsequent hosts, rapidly aid in transmission of the disease [7].

## T2S Substrates for a Dual Life Cycle: In the Human Host and in an Aquatic Niche

The T2S system is a crucial player in the fitness of *V. cholerae* during its dual lifestyle, supporting the secretion of a battery of exoproteins (Figure 1, Table S1). Some of these proteins function either in the aquatic or in the mammalian stage of the *V. cholerae* life cycle. Remarkably however, an increasing number of studies have described cargo proteins that are vital for the bacteria in both ecological niches. Among the already well-characterized dual-life-cycle proteins are the surface-exposed N-acetyl-D-glucosamine binding protein (GbpA) and a soluble Zn-metalloprotease, hemagglutinin/protease (HapA). GbpA facilitates the ability of *V. cholerae* to attach to abiotic and biotic chitinous surfaces (e.g., exoskeletons of copepods) as well as mucins covering intestinal epithelial cells (Figure 1) [8], [9]. The recently solved crystal structure and detailed molecular analysis of GbpA revealed mechanisms underlying the dual function of this four-domain protein. In particular, domains 1 and 4 are required for efficient binding to chito-oligosaccharides, and additionally domain 1 supports mucin binding. Domains 2 and 3 tether GbpA to the *V. cholerae* cell surface and together with domain 1 allow the bacteria to successfully colonize the infant mouse intestine [10]. While GbpA seems to be a broad-host colonization factor, the zinc-dependent metalloprotease HapA may be required to obtain nutrients for the bacteria in different environments encountered by *V. cholerae* during its life cycle. In particular, HapA displays proteolytic activity against a number of substrates present in the intestinal milieu of a human host including ovomucin, fibronectin, and lactoferrin [11]. In a natural reservoir, HapA degrades a gelatinous matrix covering the eggs of chironomids (Figure 1) [12], [13]. Studies in different experimental cholera models suggest that HapA is not a virulence factor *per se*, but rather it facilitates detachment of bacteria from the intestinal cells layers and increases the ability of *V. cholerae* to penetrate through the thick mucus and exit from the host [14].

Biofilm formation aids *V. cholerae* survival in both the human host and aquatic habitats [15]. A putative T2S-dependent protein, RbmC, is required together with another secreted protein, Bap1, for maintaining the structural integrity of biofilms at the air-liquid interface and on solid surfaces [16]. Whether the secretion of Bap1 is also T2S-dependent remains to be elucidated.

## T2S Exoproteins and Survival in the Aquatic Environment

The persistence of *V. cholerae* in natural habitats is a crucial factor in the epidemiology of cholera [17]. Thus a better understanding of the mechanisms that facilitate this life stage of *V. cholerae* may lead to the development of new preventative strategies for cholera epidemics. During the environmental phase of its life cycle, *V. cholerae* can exist in a free-living, planktonic form or in aggregates attached to chitinous surfaces of aquatic organisms and their eggs, or abiotic chitin particles (Figure 1) [12]. Chitin consists of multiple β-1,4-linked N-acetyl-D-glucosamine (GlcNAc) residues, and it is one of the most abundant polysaccharides in the aquatic biosphere. Many marine bacteria including *V. cholerae* contribute to the cycling of nutrients in coastal and pelagic ecosystems by utilizing chitin as a carbon and nitrogen source [17]. Chitin binding and utilization provides a substrate for *V. cholerae* multiplication and may give the vibrios protection from being killed by stomach acid during ingestion by the human host. Moreover, a significant relationship between zooplankton blooms and cholera cases has been demonstrated [17]. The T2S machinery of *V. cholerae* participates in the chitin utilization program by transporting at least six proteins, including three chitinases (ChiA-1, ChiA-2, and a putative chitinase VC0769), GbpA, chitin oligosaccharide deacetylase (COD), and spindolin-related protein (Table S1). In general, chitinases hydrolyze glycosidic bonds in chitin, releasing GlcNAc, glucosamine, and oligosaccharides that consist of either one or two of these monosaccharides. The possible different specificities and modes of action of *V. cholerae* chitin-degrading proteins have not been addressed with the exception of COD, which contributes to chitin breakdown by removing the N-acetyl group from a broad spectrum of chitin oligosaccharides [18].

## Functional Interplay between the T2S Exoproteins

T2S is responsible for the translocation of various hydrolytic enzymes that may work in concert to degrade biological material (e.g., chitin) and/or enhance pathogenicity, thus contributing to the overall fitness of *V. cholerae* in different stages of its life cycle. For instance, several T2S-dependent proteins have been implicated in aiding the function of cholera toxin (Figure 1). The “mucinase complex,” consisting of HapA and sialidase, contributes to the degradation of the epithelial mucus layer, revealing GM1 gangliosides and allowing binding and uptake of cholera toxin [19]–[21]. Additionally, genetic and biochemical analyses showed that proteases HapA, VesA, and VesB have the ability to process the proteolytically sensitive loop connecting the A_1_ and A_2_ peptide chains of cholera toxin A subunit [2], [6]. The significance of these overlapping activities of *V. cholerae* endogenous proteases is difficult to dissect *in vivo* because the host intestinal cells also supply protease(s) that activates toxin [5].

In addition to cholera toxin, HapA is also responsible for the processing and activation of the cytolytic toxin cytolysin/haemolysin A (HlyA, VCC) in some strains of *V. cholerae* [22]. The mature VCC causes induction of a chloride efflux from human intestinal epithelium by forming anion channels on the apical membranes of enterocytes, thus contributing to diarrhea [23]. Interestingly, removal of the 15-kDa fragment of pro-HlyA generating the mature VCC toxin could also be accomplished by serine proteases, and it is unknown whether the recently identified T2S-dependent proteases VesA, VesB, and VesC participate in this process.

Another fascinating example of a functional interplay between the T2S cargo proteins involves Hap and GbpA (Figure 1). As mentioned earlier, GbpA facilitates attachment of *V. cholerae* to human intestinal cells during initial stages of colonization and enhances microcolony formation [9]. Late in infection, when bacteria reach higher cell densities and vibrios are preparing for “mucosal escape,” GbpA is degraded by Hap. This process likely enhances the detachment of bacteria from the intestinal cells and facilitates their departure into the aquatic environment [24].

## Regulation of Expression of the T2S Substrates

The expression of secreted proteins remains under strict and often complex regulation. Factors contributing to the expression of cholera toxin and HapA have been intensely studied, while scarce information is available for the remaining cargo proteins. At low cell density, two regulator proteins, AphA and AphB, cooperate at the *tcpPH* promoter, leading to the increased production of TcpP and TcpH, which in concert with ToxR/S activate expression of *toxT*. Subsequently, ToxT directly activates virulence gene expression including cholera toxin. As the density of bacteria increases, a quorum-sensing master regulator HapR represses expression of cholera toxin via inhibition of transcription of *aphA* and *tcpPH* [3], [25]. HapR also negatively regulates the expression of GbpA [24]. In contrast, production of HapA remains under the positive regulation of HapR [26]. Interestingly, we have recently found a similar temporal pattern of cargo expression, with peak induction of *vesB* and *vesC* at lower cell densities (mid-logarithmic phase), while for *vesA*, the peak occurs in the stationary phase of bacterial growth (Zielke, R. A. and Sikora, A. E., unpublished data).

A number of environmental factors encountered by the bacteria in different ecological niches contribute to the production of secreted proteins. In particular, production of cholera toxin increases when *V. cholerae* is cultured anaerobically in the presence of trimethylamine N-oxide as a terminal electron acceptor—conditions that mimic the environment of the human intestine [27]. Likewise, the presence of mucin, bile salts, and nutrient limitation induce expression of *hapA* [14], [28]. Exposure to crab shell chitin and chitin oligosaccharides induces expression of genes encoding ChiA-1, ChiA-2, GbpA, and a putative chitinase (VC0769) in a manner dependent on a membrane-bound chitin-sensing histidine kinase, ChiS. Moreover, *V. cholerae* associated with living copepods strongly expresses the gene encoding spindolin-related protein (VCA0140), suggesting a role of this protein in the chitin utilization program [8].

These examples suggest that distinct exoproteins are translocated to the extracellular milieu at different stages of bacterial growth, and the mechanisms of temporal exoprotein secretion are regulated at least at the level of cargo gene transcription.

## Final Remarks

Genetic studies and proteomic approaches have broadened our appreciation of the repertoire of exoproteins secreted by the T2S system in *V. cholerae*. The contribution of this secretion pathway to *V. cholerae* pathophysiology was further underscored by findings that genetic inactivation of the T2S leads to severe pleiotropic effects, including loss of secretion and virulence, reduced bacterial growth, and induction of several stress responses [29], [30]. Thus the T2S system should be considered a prospective target for the development of new antimicrobial agents.

## Supporting Information

Table S1 Proteins secreted by the T2S and their function (or putative function) in the *V. cholerae* life cycle.
(DOCX)
