# Supplementary material for: Proteins Secreted via the Type II Secretion System: Smart Strategies of Vibrio cholerae to Maintain Fitness in Different Ecological Niches
Source: PLoS Pathog. 2013 Feb 21;9(2):e1003126. doi: 10.1371/journal.ppat.1003126 (PMC3578741; doi:10.1371/journal.ppat.1003126)
Supplement: Table S1 — Proteins secreted by the T2S and their function (or putative function) in the V. cholerae life cycle. (DOCX) [file ppat.1003126.s001.docx]

| **Gene/s** | **Name** | **Function/putative function** | **References** |
| --- | --- | --- | --- |
| VC1456-57  *ctxAB* | Cholera toxin | Primary virulence factor, induces secretory diarrhea  Required for lethal infection in *Drosophila melanogaster* | [[1](#_ENREF_1), [2](#_ENREF_2)] |
| VCA0865 | Hemagglutynin protease, HapA | Degrades mucin, fibronectin, lactoferrin Proteolytically cleaves cholera toxin and cytolysin, degrades GbpA  May facilitate “mucosal escape”  Degrades gelatinous matrix covering chironomid egg mass | [[3-9](#_ENREF_3)] |
| VC1784 | Sialidase (neuraminidase) | Removes sialic acid groups from higher order gangliosides to reveal GM1, receptor for cholera toxin  May play nutritional role by supplying sialic acid as a carbon and energy source | [[10](#_ENREF_10)] |
| VCA0219 | Cytolysin, VCC | Induces efflux of a chloride from the apical membranes of enterocytes, might contribute to the diarrhea | [[11](#_ENREF_11)] |
| VCA0811 | Glc-NAc binding protein, GbpA | Broad-host colonization factor, facilitates attachment of *V. cholerae* to chitinous surfaces and human intestinal cells | [[12-14](#_ENREF_12)] |
| VCA0812 | Leucine aminopeptidase, Lap | Not known, cleaves leucyl-p-nitroanilide, a synthetic substrate commonly used for aminopeptidases | [[15](#_ENREF_15)] |
| VCA0813 | Aminopeptidase, LapX | Not known | [[16](#_ENREF_16)] |
| VC0930 | RbmC | Together with Bap1 is responsibe for maintaining structural integrity of *V. cholerae* biofilms | [[17](#_ENREF_17)] |
| VCA0803 | Serine protease, VesA | Proteolytically processes cholera toxin A subunit | [[16](#_ENREF_16)] |
| VC1200 | Serine protease, VesB | Proteolytically cleaves cholera toxin A subunit | [[16](#_ENREF_16)] |
| VC1649 | Serine protease, VesC | Induces a haemorrhagic response in rabbit illeal loop model | [[18](#_ENREF_18)] |
| VCA0148 | TagA-related protein | Not known | [[16](#_ENREF_16)] |
| VC1952 | Chitinase ChiA-1 | Chitin utilization program | [[12](#_ENREF_12), [19](#_ENREF_19)] |
| VCA0027 | Chitinase ChiA-2 | Chitin utilization program | [[12](#_ENREF_12)] |
| VC1280 | Chitin oligosaccharide deacetylase (COD) | Chitin utilization program  Removes N-acetyl group from chitin oligosaccharides | [[12](#_ENREF_12), [20](#_ENREF_20)] |
| VC0769 | Putative chitinase | Chitin utilization program | [[12](#_ENREF_12)] |
| VCA0140 | Spindolin-related protein | Chitin utilization program | [[12](#_ENREF_12)] |
| VCA0738 | Putative uncharacterized protein | Not known | [[16](#_ENREF_16)] |
| VC2298 | Putative lipoprotein | Not known | [[16](#_ENREF_16)] |

**Table S1. Proteins secreted by the T2S and their function (or putative function) in the *V. cholerae* life cycle**

**References**

1. Kaper JB, Morris JG, Jr., Levine MM (1995) Cholera. Clin Microbiol Rev 8: 48-86.

2. Blow NS, Salomon RN, Garrity K, Reveillaud I, Kopin A, et al. (2005) *Vibrio cholerae* infection of drosophila melanogaster mimics the human disease cholera. PLoS Pathog 1: e8.

3. Robert A, Silva A, Benitez JA, Rodriguez BL, Fando R, et al. (1996) Tagging a *Vibrio cholerae* El Tor candidate vaccine strain by disruption of its hemagglutinin/protease gene using a novel reporter enzyme: *Clostridium thermocellum* endoglucanase a. Vaccine 14: 1517-1522.

4. Silva AJ, Pham K, Benitez JA (2003) Haemagglutinin/protease expression and mucin gel penetration in El Tor biotype *Vibrio cholerae*. Microbiology 149: 1883-1891.

5. Broza M, Halpern M (2001) Pathogen reservoirs. Chironomid egg masses and *Vibrio cholerae*. Nature 412: 40.

6. Halpern M, Gancz H, Broza M, Kashi Y (2003) *Vibrio cholerae* hemagglutinin/protease degrades chironomid egg masses. Appl Environ Microbiol 69: 4200-4204.

7. Booth BA, Boesman-Finkelstein M, Finkelstein RA (1983) *Vibrio cholerae* soluble hemagglutinin/protease is a metalloenzyme. Infect Immun 42: 639-644.

8. Finkelstein RA, Boesman-Finkelstein M, Holt P (1983) *Vibrio cholerae* hemagglutinin/lectin/protease hydrolyzes fibronectin and ovomucin: F.M. Burnet revisited. Proc Natl Acad Sci U S A 80: 1092-1095.

9. Jude BA, Martinez RM, Skorupski K, Taylor RK (2009) Levels of the secreted *Vibrio cholerae* attachment factor GbpA are modulated by quorum-sensing-induced proteolysis. J Bacteriol 191: 6911-6917.

10. Galen JE, Ketley JM, Fasano A, Richardson SH, Wasserman SS, et al. (1992) Role of *Vibrio cholerae* neuraminidase in the function of cholera toxin. Infect Immun 60: 406-415.

11. Debellis L, Diana A, Arcidiacono D, Fiorotto R, Portincasa P, et al. (2009) The *Vibrio cholerae* cytolysin promotes chloride secretion from intact human intestinal mucosa. PLoS ONE 4: e5074.

12. Meibom KL, Li XB, Nielsen AT, Wu CY, Roseman S, et al. (2004) The *Vibrio cholerae* chitin utilization program. Proc Natl Acad Sci U S A 101: 2524-2529.

13. Kirn TJ, Jude BA, Taylor RK (2005) A colonization factor links *Vibrio cholerae* environmental survival and human infection. Nature 438: 863-866.

14. Wong E, Vaaje-Kolstad G, Ghosh A, Hurtado-Guerrero R, Konarev PV, et al. (2012) The *Vibrio cholerae* colonization factor GbpA possesses a modular structure that governs binding to different host surfaces. PLoS Pathog 8: e1002373.

15. Toma C, Honma Y (1996) Cloning and genetic analysis of the *Vibrio cholerae* aminopeptidase gene. Infect Immun 64: 4495-4500.

16. Sikora AE, Zielke RA, Lawrence DA, Andrews PC, Sandkvist M (2011) Proteomic analysis of the *Vibrio cholerae* type II secretome reveals new proteins, including three related serine proteases. J Biol Chem 286: 16555-16566.

17. Fong JC, Yildiz FH (2007) The *rbmbcdef* gene cluster modulates development of rugose colony morphology and biofilm formation in *Vibrio cholerae*. J Bacteriol 189: 2319-2330.

18. Syngkon A, Elluri S, Koley H, Rompikuntal PK, Saha DR, et al. Studies on a novel serine protease of a Δ*hapA*Δ*prtv* *Vibrio cholerae* O1 strain and its role in hemorrhagic response in the rabbit ileal loop model. PLoS ONE 5:

19. Connell TD, Metzger DJ, Lynch J, Folster JP (1998) Endochitinase is transported to the extracellular milieu by the *eps*-encoded general secretory pathway of *Vibrio cholerae*. J Bacteriol 180: 5591-5600.

20. Li X, Wang LX, Wang X, Roseman S (2007) The chitin catabolic cascade in the marine bacterium *Vibrio cholerae*: Characterization of a unique chitin oligosaccharide deacetylase. Glycobiology 17: 1377-1387.
